# Black, Asian and Minority Ethnic groups in England are at increased risk of death from COVID-19: indirect standardisation of NHS mortality data

**DOI:** 10.12688/wellcomeopenres.15922.2

**Published:** 2020-06-24

**Authors:** Robert W. Aldridge, Dan Lewer, Srinivasa Vittal Katikireddi, Rohini Mathur, Neha Pathak, Rachel Burns, Ellen B. Fragaszy, Anne M. Johnson, Delan Devakumar, Ibrahim Abubakar, Andrew Hayward

**Affiliations:** 1 UCL Public Health Data Science Research Group, Institute of Health Informatics, UCL, London, NW1 2DA, UK; 2 UCL Collaborative Centre for Inclusion Health, Institute of Epidemiology and Health Care, UCL, London, WC1E 7HB, UK; 3 MRC/CSO Social & Public Health Sciences Unit, University of Glasgow, London, G2 3AX, UK; 4 Department of Non-Communicable Disease Epidemiology, London School of Hygiene and Tropical Medicine, London, WC1E 7HT, UK; 5 Guy’s and St Thomas’s NHS Foundation Trust, London, SE1 9RT, UK; 6 Department of Infectious Disease Epidemiology, London School of Hygiene and Tropical Medicine, London, WC1E 7HT, UK; 7 UCL Institute for Global Health, London, WC1N 1EH, UK

**Keywords:** SARS-CoV-2, COVID-19, Mortality, minority ethnic groups

## Abstract

**Background**: International and UK data suggest that Black, Asian and Minority Ethnic (BAME) groups are at increased risk of infection and death from COVID-19. We aimed to explore the risk of death in minority ethnic groups in England using data reported by NHS England.

**Methods**: We used NHS data on patients with a positive COVID-19 test who died in hospitals in England published on 28th April, with deaths by ethnicity available from 1st March 2020 up to 5pm on 21 April 2020. We undertook indirect standardisation of these data (using the whole population of England as the reference) to produce ethnic specific standardised mortality ratios (SMRs) adjusted for age and geographical region.

**Results**: The largest total number of deaths in minority ethnic groups were Indian (492 deaths) and Black Caribbean (460 deaths) groups. Adjusting for region we found a lower risk of death for White Irish (SMR 0.52; 95%CIs 0.45-0.60) and White British ethnic groups (0.88; 95%CIs 0.86-0.0.89), but increased risk of death for Black African (3.24; 95%CIs 2.90-3.62), Black Caribbean (2.21; 95%CIs 2.02-2.41), Pakistani (3.29; 95%CIs 2.96-3.64), Bangladeshi (2.41; 95%CIs 1.98-2.91) and Indian (1.70; 95%CIs 1.56-1.85) minority ethnic groups.

**Conclusion: **Our analysis adds to the evidence that BAME people are at increased risk of death from COVID-19 even after adjusting for geographical region, but was limited by the lack of data on deaths outside of NHS settings and ethnicity denominator data being based on the 2011 census. Despite these limitations, we believe there is an urgent need to take action to reduce the risk of death for BAME groups and better understand why some ethnic groups experience greater risk. Actions that are likely to reduce these inequities include ensuring adequate income protection, reducing occupational risks, reducing barriers in accessing healthcare and providing culturally and linguistically appropriate public health communications.

## Background

There is increasing international evidence that Black, Asian and Minority Ethnic (BAME) people are at higher risk of death from COVID-19 ^1^. As of 26th April 2020, there were 20,732 reported COVID-19 deaths in hospital in the UK ^2^, but to date, there have been no officially reported analyses of the risk of death by ethnicity ^3^. An inquiry has been announced that will examine the impact of COVID-19 on BAME people ^4^.

Ethnicity data are currently available in the intensive care national audit and research centre (ICNARC) reports on patients with confirmed COVID-19 that have been admitted to intensive care for at least 24 hours. On 24th April 2020 these data showed that BAME people were at higher risk of developing severe COVID-19 disease ^5^. A total of 5,993 patients with confirmed COVID-19 had reported data on ethnicity and 34.2% (2,055/5,993) of these patients were from BAME groups. Analyses matched by area (ward) of residence showed differences are significant for all BAME groups but there is substantial variation by minority ethnic groups. There were 1.63 times more Black patients in critical care than expected based on the matched population (10.6% vs 6.5%). For Asian patients the differential is reduced but still significant with 1.25 times more Asian patients than expected (15.3% vs 12.2%).

Ethnicity is not recorded in death certificates in England which is an important limitation on our ability to study the differential impact of COVID-19 on mortality in different BAME groups. However, daily NHS hospital death data are provided by geographical region, age and ethnicity ^6^. Adjusting for region is potentially important because in England COVID-19 has affected different parts of the country to a different extent. For example, London and the West Midlands, the two regions with the highest levels of BAME residents have had most COVID-19 cases. Using these data, we aimed to examine the risk of death from COVID-19 by BAME group and through a sensitivity analysis test whether differences between BAME groups could be explained by regional differences in the ethnic make-up of the population.

## Methods

We used NHS data on patients with a positive COVID-19 test who died in hospitals in England, in separate tables by age group, region, and ethnicity. We used data published on 26th April, that included deaths by ethnicity from 1 March 2020 up to 5pm 21 April 2020 ^6^. Where the age group and region tables showed a different total number of deaths to the ethnicity table, we applied a scaling factor to align the totals to the ethnicity table. We assumed that decedents with unknown ethnicity had the same ethnicity structure as other decedents.

We used indirect standardisation to calculate standardised mortality ratios (SMRs) by ethnic group (where the reference group is the whole population). We first calculated age-specific mortality rates using the COVID-19 deaths data and population estimates from the UK Census 2011. All ages were used, and age ranges included were 0–19; 20–39;40–59; 60–79 and 80+. We then calculated an expected number of deaths for each ethnic group by applying these mortality rates to population estimates by both ethnic group and age, also from the UK Census 2011. We calculated the SMR as the observed deaths divided by the expected deaths. We assumed that deaths occurred over the same time period for all ethnic groups, and used the population point estimate as the denominator for simplicity. We then conducted a sensitivity analysis to account for regional differences in the ethnicity of the population. The number of COVID-19 deaths by age and region was not available, and we assumed that the proportion of deaths in each age group was the same across regions. We calculated age- and region-specific mortality rates using this assumption, and calculated an expected number of deaths by applying these rates to population estimates by ethnic group, age, and region. The data included seven regions: London, South East, South West, Midlands, East of England, North West, and North East & Yorkshire and the Humber. We then estimated SMRs adjusted for region by dividing the observed by the expected deaths. We calculated 95% confidence intervals using the exact Poisson method. All analyses were conducted using R version 3.6.2. Data and code required for replication are provided as Underlying and Extended data ^7^.

## Results

A total of 16,272 deaths were observed over the study period. Ethnicity was missing for 9.4% (1,537/16,272) of NHS England hospital deaths. The largest total number of deaths in minority ethnic groups were Indian (492 deaths) and Black Caribbean (460 deaths) people. In comparison to the whole population, SMRs in the unadjusted analysis were reduced for White British and White Irish groups, but were increased for all BAME groups. After adjusting for region, White Irish (SMR 0.52; 95%CIs 0.45-0.60) and White British (SMR 0.88; 95%CIs 0.86-0.89) ethnic groups continued to have a lower risk of death. Black African (3.24; 95%CIs 2.90-3.62), Black Caribbean (2.21; 95%CIs 2.02-2.41), Pakistani (3.29; 95%CIs 2.96-3.64), Bangladeshi (2.41; 95%CIs 1.98-2.91) and Indian (1.70; 95%CIs 1.56-1.85) minority ethnic groups continued to have a higher risk of death ( Table 1). There was no statistical evidence that SMRs were increased or reduced for Chinese (1.14; 95%CIs 0.87-1.45), Mixed White and Black African (1.31; 95%CIs 0.70-2.25), Mixed White and Asian (0.93; 95%CIs 0.59-1.38) and Mixed White and Black Caribbean (1.10; 95%CIs 0.77-1.53) ethnic groups.

**Table 1.** NHS England COVID-19 deaths by ethnic group, adjusted and unadjusted by NHS region (Numbers in brackets show 95% confidence intervals).

Group	Subgroup	Observed deaths *	Expected deaths (adjusting for age)	SMR (adjusting for age)	p-value (adjusting for age)	Expected deaths (adjusting for age and region)	SMR (adjusting for age and region)	p-value (adjusting for age and region)
Asian	Chinese	63	40.1	1.57 (1.21-2.01)	<0.001	55.4	1.14 (0.87-1.45)	0.313
	Bangladeshi	110	27.7	3.97 (3.26-4.79)	<0.001	45.6	2.41 (1.98-2.91)	<0.001
	Asian other	271	78.9	3.44 (3.04-3.87)	<0.001	125.9	2.15 (1.90-2.42)	<0.001
	Pakistani	367	91	4.03 (3.63-4.47)	<0.001	111.6	3.29 (2.96-3.64)	<0.001
	Indian	543	208.1	2.61 (2.39-2.84)	<0.001	319.3	1.70 (1.56-1.85)	<0.001
	Total: Asian	1,354	445.8	3.04 (2.88-3.20)	<0.001	657.8	2.06 (1.95-2.17)	<0.001
Black	Black other	161	18.5	8.70 (7.41-10.15)	<0.001	30.8	5.23 (4.46-6.11)	<0.001
	African	320	56.3	5.68 (5.08-6.34)	<0.001	98.6	3.24 (2.90-3.62)	<0.001
	Caribbean	508	136.9	3.71 (3.39-4.05)	<0.001	230.3	2.21 (2.02-2.41)	<0.001
	Total: Black	989	211.8	4.67 (4.38-4.97)	<0.001	359.7	2.75 (2.58-2.93)	<0.001
Mixed	White and Black African	13	7.0	1.86 (0.99-3.18)	0.034	9.9	1.31 (0.70-2.25)	0.335
	White and Asian	24	19.7	1.22 (0.78-1.81)	0.311	25.9	0.93 (0.59-1.38)	0.844
	White and Black Caribbean	36	26.4	1.36 (0.95-1.89)	0.064	32.6	1.10 (0.77-1.53)	0.539
	Mixed other	53	19.2	2.76 (2.07-3.61)	<0.001	26.8	1.98 (1.48-2.59)	<0.001
	Total: Mixed	126	72.3	1.74 (1.45-2.07)	<0.001	95.2	1.32 (1.10-1.58)	0.002
Other	Total: Other	485	53.5	9.06 (8.27-9.90)	<0.001	86.4	5.62 (5.13-6.14)	<0.001
White	White Irish	178	262.8	0.68 (0.58-0.78)	<0.001	342	0.52 (0.45-0.60)	<0.001
	White other	601	333.3	1.80 (1.66-1.95)	<0.001	445.6	1.35 (1.24-1.46)	<0.001
	White British	12,538	14,892.5	0.84 (0.83-0.86)	<0.001	14,285.40	0.88 (0.86-0.89)	<0.001
	Total: White	13,317	15,488.6	0.86 (0.85-0.87)	<0.001	15,073.00	0.88 (0.87-0.90)	<0.001

* Ethnicity was missing for 1,537 deaths. We assumed that decedents with unknown ethnicity had the same ethnicity structure as other decedents and redistributed these deaths between ethnic groups. As a result, the numbers of deaths by minority ethnic group do not match those reported in the abstract and results section which presents numbers from NHS Data by ethnicity before this redistribution. CI=Confidence Interval SMR= Standardised Mortality Ratio

## Discussion

Our analyses showed that several BAME groups have a higher risk of death from COVID-19 and that regional differences in ethnicity explains some but not all of the differences between ethnic groups. After accounting for geographical region SMRs reduced, but there remained large differences in SMRs between ethnic groups - White British and White Other have lower SMRs, but Bangladeshi, Pakistani, Indian, Black African and Black Caribbean ethnic groups all have substantially increased SMRs.

There are limitations to these data relating to the reporting of COVID-19 deaths. The NHS data we have used are currently only available in broad age groups, and are not broken down by both region and age, which meant we had to assume there were no differences in age structure of deaths across regions within these age bands. Data are also not disaggregated by sex and social deprivation and therefore we were unable to explore the effect these would have on our adjusted SMR estimates. There is increasing evidence that men are more likely to die from COVID-19 and therefore our lack of disaggregation by sex could account for some of the remaining differences in SMRs we see between ethnic groups, particularly as those occupations found to be at higher risk include greater numbers of men working in them. Publication of COVID-19 death data by age, region, gender, social deprivation, and ethnicity would improve these adjustments further. The data we used only include people who died in hospital. ONS data from 28 April suggest that 77.4% (14,796 deaths) of all deaths in England and Wales occurred in hospital with 3,096 deaths occurring in care homes ^8^ and the SMRs may be biased if deaths that occur in hospitals differ from those occurring in the community by ethnic group, which might arise due to, for example, differences in use of residential care homes ^9^. Deaths in residential care homes are likely to include a larger number of White British people ^10^ which could lead to an under-estimation of the SMR in this group within our estimates. Our analysis was based on the 2011 census data and therefore will not reflect recent changes in the age, ethnic and region across England in the last nine years. Our use of census data from 2011 is likely to result in over-estimation of mortality ratios in minority ethnic groups that have grown the fastest during this time period. We found raised SMRs in several BAME groups including Asian Other, Black Other, Mixed Other and White Other and further analysis should be undertaken to examine whether there are particular groups at risk within these broad groups to ensure we can better understand their increased mortality risk.

Our analysis is consistent with Intensive Care National Audit and Research Centre (ICNARC) data which suggests that Black ethnic groups are substantially over-represented amongst critical care patients, and that BAME groups in critical care are generally more likely to require ventilation and therefore more likely to die. Further analyses of the ICNARC data are required to assess the extent to which these associations are due to differences in age, comorbidity and socioeconomic status. A recent analysis of COVID-19 deaths in health and social care workers was undertaken using data from mainstream and social media reports. This analysis suggested that BAME deaths in nursing and support staff accounted for 64% of deaths, and 95% in medical staff, whilst these groups accounted for 20% and 44% percent of all staff ^11^. This analysis of deaths in health and social workers, however, did not adjust for regional differences in the proportion of NHS staff coming from BAME groups.

**Figure 1.**
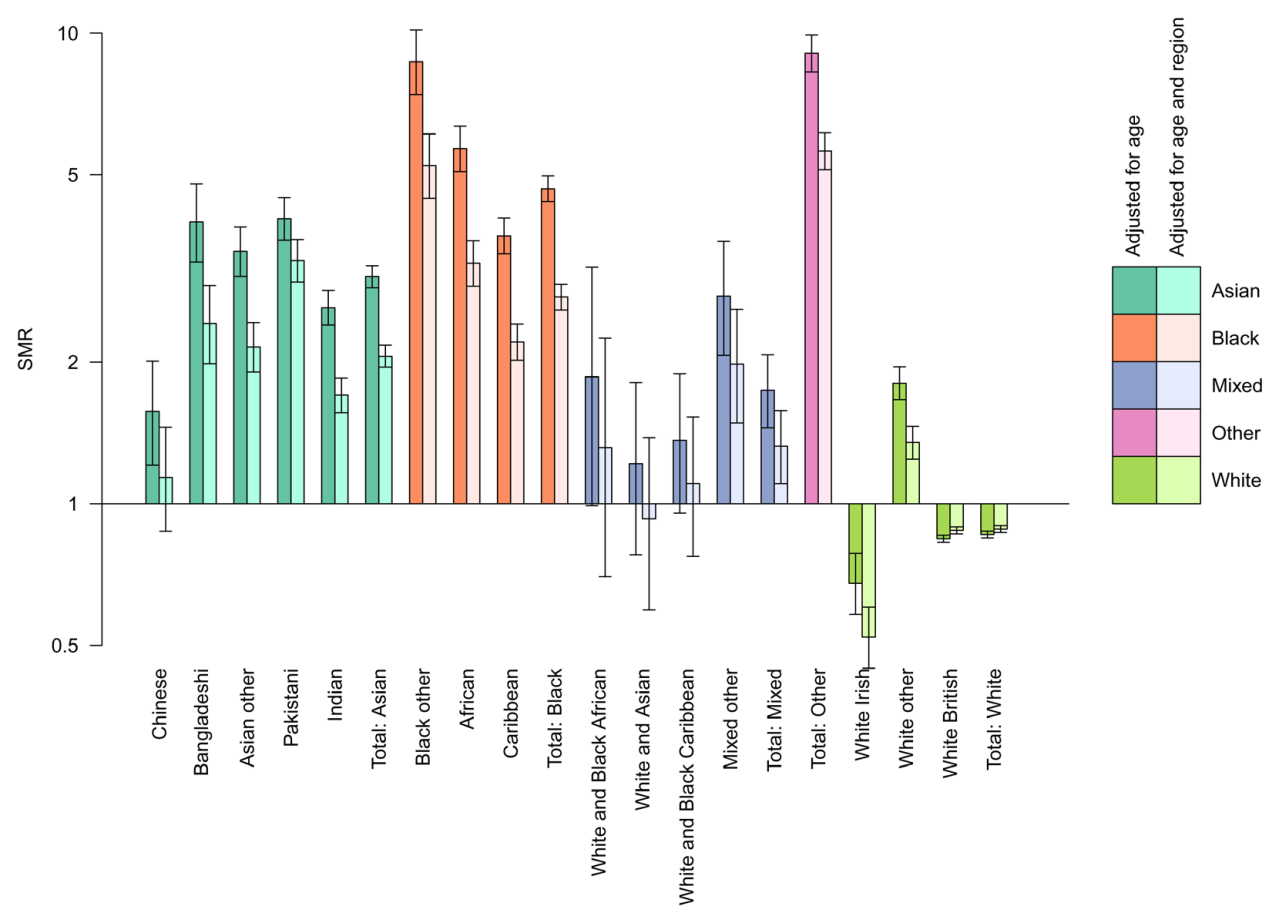
NHS England COVID-19 deaths by ethnic group, adjusted and unadjusted by NHS region (error bars show 95% confidence intervals).

Adjusting for geographic region reduced the high SMRs for BAME groups as shown in Figure 1. Several other factors, some of which will be associated with geographic region, may further explain this increased risk. There is increasing evidence from ONS and Public Health England on the role of occupation and socio-economic deprivation in relation to risk of COVID-19 and the increased risk of infection and poor outcomes found in BAME communities ^12– 15^. Occupation is also likely to play an important role in terms of increased risk of infection as BAME people are more likely to have occupations that involve greater social mixing and less ability to work from home. For example, Black groups are overrepresented in caring and leisure industries; Pakistani and Bangladeshi groups are overrepresented in sales and consumer service occupations; and Black groups in public administration, education and health ^16^. BAME groups are more likely to have a low income, be in zero hours contracts and non salaried jobs than white ethnic groups. This may make it harder to comply with social distancing restrictions that prevent people from working and those who are self-employed or working in the gig economy will have their earnings stop unless they sign up to a government scheme. There may be barriers to this and some migrants, for example, may not want to make themselves known to the authorities. Ethnicity is socially constructed and correlates poorly with biology. Biological differences are therefore highly unlikely to underpin these inequalities ^17^.

Living in overcrowded housing likely increases transmission risk, and BAME households were more likely to be overcrowded than White British households in recent analysis by ONS ^18^. This is true even when restricting analyses to those living in poverty, where BAME groups living in poverty are more likely to be in overcrowded conditions than white groups living in poverty ^19^. Increased levels of pre-existing medical conditions such as diabetes, hypertension and heart disease are known to increase the risk of severe COVID-19 disease ^1^ and these are also increased in some ethnic groups. Finally, differences in risk factors such as obesity, may also be relevant. Research to disentangle these potential pathways appears highly limited, with only one study having been conducted, to our knowledge ^20^. This was based on laboratory-confirmed diagnoses using the UK Biobank study and suggested that socioeconomic differences might make an important contribution, but differences in pre-existing health and risk factors appeared less important. However, this study was based on a non-representative sample and relied on routine testing which is likely subject to substantial ascertainment bias.

Ethnicity is not recorded in death certificates in England, which is a major limitation in our ability to study the differential impact of COVID-19 on mortality in different ethnic groups. However, this has been achieved in Scotland and the COVID-19 pandemic highlights the potential utility of introducing it in England ^21^. Further analysis of deaths for BAME people will require urgent linkage to other records that contain ethnicity information such as NHS hospital episode statistics and primary care electronic health records. A key unanswered question is to understand why mortality risks differ between ethnic groups. This may arise from an increased risk of developing infection, worse prognosis or care once infection has occurred or a combination of the above ^22^. While it is important to conduct and report such analyses rapidly, this must not delay immediate action to begin to mitigate these extreme inequities.

We believe there are several important and urgent public health actions to be taken to address the high mortality rates in BAME groups described in our analyses. First, some BAME groups face barriers in accessing high quality healthcare. The NHS must remove these barriers working with minority ethnic people to understand the issues. For example, some people in BAME communities will also be international migrants and Public Health England recently reported the increased risk of death from COVID-19 in this group ^15^. This analysis showed that the largest relative increase in death from COVID-19 was for people born in Central and Western Africa, the Caribbean, South East Asia, the Middle East and South and Eastern Africa. Some groups of international migrants in the UK avoid the use of the NHS because of the current NHS charging regime for migrants or through fear of their data being shared with the Home Office for immigration enforcement purposes ^23^. Limited healthcare entitlement results in untreated conditions, poorly managed chronic conditions and deterrence from healthcare for migrants is well documented, rendering a context of distrust and fear ^24^. Whilst migrants diagnosed with COVID-19 are exempt from healthcare charges, not all migrants will be aware of these exemptions and the exemption first requires a diagnosis. Some migrants may fear the charge being imposed through a lack of diagnosis due to limited testing opportunities. We therefore call for the removal of all NHS charges during this public health emergency to ensure that no migrant or individual from a BAME group delays seeking healthcare and risks death through fear of being charged for their NHS care. Second, we must ensure that linguistically and culturally appropriate public health communication and engagement is being provided and appropriately targeted at those populations at greatest risk. This needs to be developed with affected communities and tailored to specific challenges including addressing culturally specific disinformation and, for example, addressing the difficulties of preventing transmission in overcrowded households or of shielding vulnerable people in multigenerational households. Third, we must take urgent action to reduce the risk of SARS-CoV-2 infection in BAME populations. For example, BAME groups are more likely to work in care settings such as nursing homes, where adequate PPE to prevent infection is vital. BAME groups are also more likely than others to be in key worker occupational groups who have high levels of exposure to the general public and therefore high risk of infection. The effectiveness of personal protective equipment in preventing infection outside health and social care settings remains uncertain, however, there are a range of other measures that are likely to reduce infection risk. These include: ensuring that workplaces are not overcrowded so that staff can maintain social distancing at work; providing distancing measures and physical barriers to reduce exposure to droplets from the members of the general public (e.g. perspex screens at supermarket counters); ready availability of handwashing materials at the workplace and access to testing and workplace contact tracing. Fourth, there is a risk that some ethnic minority groups might not only experience greater risks from COVID-19 itself, but also greater adverse consequences of the extensive social distancing measures in place ^25^. There is a need for adequate income protection to ensure low paid, non-salaried and zero-hours contract workers can afford to follow isolation and “stay at home” recommendations.

The unacceptable differences in COVID mortality between white and BAME groups demand immediate action. They are an extreme example of the long-standing inequities affecting BAME groups in our society. As we emerge from the COVID-19 pandemic we must ensure that these unfair and avoidable disparities are addressed. Governments in the UK, and elsewhere, must consider how to best protect minority ethnic groups from experiencing further disadvantage and indirect health harms during the recovery process. The public health response to COVID-19 must be equitable and urgent if it is to address the unacceptable ethnic disparities our analyses show.

## Data availability

### Source data

Source data for this analysis comes from the NHS COVID-19 Daily Deaths

https://www.england.nhs.uk/statistics/statistical-work-areas/covid-19-daily-deaths/ which we have used and reproduced under the terms of the Open Government Licence v3.0.

### Underlying data

UCL Discovery: Dataset: Black, Asian and Minority Ethnic groups in England are at increased risk of death from COVID-19: indirect standardisation of NHS mortality data. https://doi.org/10.14324/000.ds.10096589 ^7^

This project contains the following underlying data:

- lookup_region.csv (Look up table to align region categories in Census population data with NHSE data)
- nomis_reformatted.csv (2011 Census population data)
- region_26apr2020.csv (NHSE COVID-19 death data by region)
- age_26apr2020.csv (NHSE COVID-19 death data by age)
- ethnicity_26apr2020 (NHSE COVID-19 death data by ethnicity)
- lookup_age.csv (Look up table to align age categories in Census population data with NHSE data)
- lookup_ethnicity.csv (Look up table to align ethnicity categories in Census population data with NHSE data)

### Extended data

UCL Discovery: UCL Discovery: Dataset: Black, Asian and Minority Ethnic groups in England are at increased risk of death from COVID-19: indirect standardisation of NHS mortality data. https://doi.org/10.14324/000.ds.10096589 ^7^

This project contains the following extended data:

- Aldridge_covid19_ethnicity_smrs_v4_1 (Analysis replication code)

Data are available under the terms of the Creative Commons Attribution 4.0 International license (CC-BY 4.0).
