# Supplementary material for: Black, Asian and Minority Ethnic groups in England are at increased risk of death from COVID-19: indirect standardisation of NHS mortality data
Source: Wellcome Open Res. 2020 Jun 24;5:88. Originally published 2020 May 6. [Version 2] doi: 10.12688/wellcomeopenres.15922.2 (PMC7317462; doi:10.12688/wellcomeopenres.15922.2)
Supplement: Peer review history

# Peer review: Reviewer response for version 2

**DOI:** 10.21956/wellcomeopenres.17630.r39222

**Published:** 2020-08-27

**Authors:** Matthew Wallace

**Affiliations:** 1 Demography Unit, Department of Sociology, Stockholm University, Stockholm, Sweden

The authors have responded in detail to each of my comments and addressed the most pressing ones. As of now, the revised version of this interesting manuscript has satisfied the previous reservations I had.

Is the work clearly and accurately presented and does it cite the current literature?

Yes

If applicable, is the statistical analysis and its interpretation appropriate?

Yes

Are all the source data underlying the results available to ensure full reproducibility?

Yes

Is the study design appropriate and is the work technically sound?

Partly

Are the conclusions drawn adequately supported by the results?

Yes

Are sufficient details of methods and analysis provided to allow replication by others?

Yes

Reviewer Expertise:

Demography, Population Studies, Geography.

I confirm that I have read this submission and believe that I have an appropriate level of expertise to confirm that it is of an acceptable scientific standard.

# Peer review: Reviewer response for version 2

**DOI:** 10.21956/wellcomeopenres.17630.r39223

**Published:** 2020-06-29

**Authors:** Frances Darlington-Pollock

**Affiliations:** 1 Geography and Planning, University of Liverpool, Liverpool, UK

The additional clarification is welcome, as are the clearer signposts to the data used. The revisions have addressed my previous reservations.

Is the work clearly and accurately presented and does it cite the current literature?

Yes

If applicable, is the statistical analysis and its interpretation appropriate?

Yes

Are all the source data underlying the results available to ensure full reproducibility?

Partly

Is the study design appropriate and is the work technically sound?

Yes

Are the conclusions drawn adequately supported by the results?

Partly

Are sufficient details of methods and analysis provided to allow replication by others?

Yes

Reviewer Expertise:

Population Geography, Health Geography, Social and Spatial Inequality, Ethnicity.

I confirm that I have read this submission and believe that I have an appropriate level of expertise to confirm that it is of an acceptable scientific standard.

# Peer review: Reviewer response for version 2

**DOI:** 10.21956/wellcomeopenres.17630.r39221

**Published:** 2020-06-25

**Authors:** Oliver Razum, Odile Sauzet

**Affiliations:** 1 Department of Epidemiology & International Public Health, School of Public Health, Bielefeld University, Bielefeld, Germany

Our reservations have been taken care of adequately. We approve the manuscript as it now stands.

Is the work clearly and accurately presented and does it cite the current literature?

Yes

If applicable, is the statistical analysis and its interpretation appropriate?

Yes

Are all the source data underlying the results available to ensure full reproducibility?

Yes

Is the study design appropriate and is the work technically sound?

Yes

Are the conclusions drawn adequately supported by the results?

Partly

Are sufficient details of methods and analysis provided to allow replication by others?

Yes

Reviewer Expertise:

epidemiology; public health; health of migrants and refugees; small area differentials in health; Covid-19

We confirm that we have read this submission and believe that we have an appropriate level of expertise to confirm that it is of an acceptable scientific standard.

# Peer review: Reviewer response for version 1

**DOI:** 10.21956/wellcomeopenres.17464.r38665

**Published:** 2020-05-28

**Authors:** Frances Darlington-Pollock

**Affiliations:** 1 Geography and Planning, University of Liverpool, Liverpool, UK

Public and media attention has increasingly centred on the apparent disproportionate impact of COVID-19 on BAME groups. This paper adds empirical evidence to these discussions, going some way in highlighting that COVID-19 is far from a leveller or equalizer. The authors use publicly available daily NHS hospital death data provided by geographical region, age and ethnicity to create standardised mortality ratios (SMRs) by ethnic group which are first adjusted by age and then also by region (having looked at the data, the age groups are quite broad which may be worth stating). The indirect method of standardisation is used which is appropriate given the possibility of small numbers in some cases. Results are presented in a table and in a figure, illustrating that Asian (bar Chinese), Black, Mixed Other, and White Other ethnic groups all have a heightened risk of mortality from COVID-19 after adjusting for age and region. The authors go on to speculate as to possible reasons for the differences between ethnic groups, ranging from differences in experiences of poverty between regions and ethnic groups, differences in occupation and the resulting risk of exposure, housing situation, and differences in pre-existing medical conditions. The authors make a number of recommendations in terms of public health interventions during this crisis, as well as noting limitations. Though well written, clearly presented, and informative, there are a couple of factors that would strengthen this paper, particularly given the unavoidable limitations of the data used. 

1. In the recommendations for intervention section, much is made of the barriers to healthcare access faced by some BAME groups. More evidence to support this would strengthen. Further, though concerns are rightly raised as to the impact of the NHS charging regime for migrants and general discussion of migrants and differential healthcare needs/access requirements, this does not feel well supported by the data used. These data are simply ‘ethnicity’, rather than migrant status, country of birth or length of residence in the UK – all of which matter for this theme of discussion. More reflection of this needed.
2. Relatedly, much is made of how differences in occupation and accordingly, socioeconomic position, matter for risk of exposure and the wider impact of COVID-19 policy responses. These are relevant and important debates, but are tangential to what is shown in the data due to the limitations of what is recorded. More set up/evidence as to why these will vary between ethnic groups, and the importance of region (which is a rather crude geographical scale and more could be made of this), would help counter this limitation. 
3. Though not possible to produce SMRs by sex, more reflection on that is important given wider differences in COVID-19 mortality profiles between sexes, and the relationship with some of the discussion on occupation/socioeconomic factors etc. 
4. Finally (minor point): as an open piece of research this is very useful. Yet, when looking through the link provided for the data I could only see deaths broken down by region, broad age group, and NHS Trust. More specifics as to the location of the ethnicity data would be welcome (NB, the authors provide the R code and underlying data, which is great).

Is the work clearly and accurately presented and does it cite the current literature?

Yes

If applicable, is the statistical analysis and its interpretation appropriate?

Yes

Are all the source data underlying the results available to ensure full reproducibility?

Partly

Is the study design appropriate and is the work technically sound?

Yes

Are the conclusions drawn adequately supported by the results?

Partly

Are sufficient details of methods and analysis provided to allow replication by others?

Yes

Reviewer Expertise:

Population Geography, Health Geography, Social and Spatial Inequality, Ethnicity.

I confirm that I have read this submission and believe that I have an appropriate level of expertise to confirm that it is of an acceptable scientific standard, however I have significant reservations, as outlined above.

# Response

**Published:** 2020-06-15

**Authors:** Robert Aldridge

**Affiliations:** UCL, UK

Thanks very much for this helpful review - we appreciate the time you have taken to carefully read and provide constructive feedback on the article. We have tried to address all the points you have raised and below we include your comments alongside in our responses ***in bold italics. ***We have numbered each of our responses in order that we can cross-reference them across all of the reviews to this article.

  

******

Public and media attention has increasingly centred on the apparent disproportionate impact of COVID-19 on BAME groups. This paper adds empirical evidence to these discussions, going some way in highlighting that COVID-19 is far from a leveller or equalizer. The authors use publicly available daily NHS hospital death data provided by geographical region, age and ethnicity to create standardised mortality ratios (SMRs) by ethnic group which are first adjusted by age and then also by region (having looked at the data, the age groups are quite broad which may be worth stating). The indirect method of standardisation is used which is appropriate given the possibility of small numbers in some cases. Results are presented in a table and in a figure, illustrating that Asian (bar Chinese), Black, Mixed Other, and White Other ethnic groups all have a heightened risk of mortality from COVID-19 after adjusting for age and region. The authors go on to speculate as to possible reasons for the differences between ethnic groups, ranging from differences in experiences of poverty between regions and ethnic groups, differences in occupation and the resulting risk of exposure, housing situation, and differences in pre-existing medical conditions. The authors make a number of recommendations in terms of public health interventions during this crisis, as well as noting limitations. Though well written, clearly presented, and informative, there are a couple of factors that would strengthen this paper, particularly given the unavoidable limitations of the data used.

In the recommendations for intervention section, much is made of the barriers to healthcare access faced by some BAME groups. More evidence to support this would strengthen. Further, though concerns are rightly raised as to the impact of the NHS charging regime for migrants and general discussion of migrants and differential healthcare needs/access requirements, this does not feel well supported by the data used. These data are simply ‘ethnicity’, rather than migrant status, country of birth or length of residence in the UK – all of which matter for this theme of discussion. More reflection of this needed.

***Author response 9: We agree that not all people in minority ethnic groups are migrants, but there is a large overlap between these groups and we feel that the particular issues faced by migrants are important to highlight along with potential important measures to reduce their increased risk of death from COVID-19. Public Health England has produced additional evidence on this point since our publication on this increased risk for migrants and we have now added the following sentence to qualify the points we make on migration in order to address this important issue raised by the reviewer:***

***“For example, some people in BAME communities will also be international migrants and Public Health England recently reported the increased risk of death from COVID-19 in this group. This analysis showed that the largest relative increase in death from COVID-19 was for people born in Central and Western Africa, the Caribbean, South East Asia, the Middle East and South and Eastern Africa.”***

Relatedly, much is made of how differences in occupation and accordingly, socioeconomic position, matter for risk of exposure and the wider impact of COVID-19 policy responses. These are relevant and important debates, but are tangential to what is shown in the data due to the limitations of what is recorded. More set up/evidence as to why these will vary between ethnic groups, and the importance of region (which is a rather crude geographical scale and more could be made of this), would help counter this limitation.

***Author response 10: After our study was published ONS and Public Health England have completed several analyses that support the comments we make in this discussion. We have edited out some of our previous comments on these issues and replaced it with the following sentences in the discussion which provide direct evidence on the associations between occupation, socioeconomic position and COVID:***

***“There is increasing evidence from ONS and Public Health England on the role of occupation and socio-economic deprivation in relation to risk of COVID-19 and the increased risk of infection and poor outcomes found in BAME communities”***

Though not possible to produce SMRs by sex, more reflection on that is important given wider differences in COVID-19 mortality profiles between sexes, and the relationship with some of the discussion on occupation/socioeconomic factors etc.

***Author response 11: We have attempted to address this and a similar point raised by Matthew Wallace in our “Author response 2”***

Finally (minor point): as an open piece of research this is very useful. Yet, when looking through the link provided for the data I could only see deaths broken down by region, broad age group, and NHS Trust. More specifics as to the location of the ethnicity data would be welcome (NB, the authors provide the R code and underlying data, which is great).

***Author response 12: Our updated code includes links to the data sources and metadata including links to these sources which we hope is more helpful in understanding where these data come from and how we broke them down.***

# Peer review: Reviewer response for version 1

**DOI:** 10.21956/wellcomeopenres.17464.r38681

**Published:** 2020-05-26

**Authors:** Oliver Razum, Odile Sauzet

**Affiliations:** 1 Department of Epidemiology & International Public Health, School of Public Health, Bielefeld University, Bielefeld, Germany

Preliminary reports indicate that Black, Asian and Minority Ethnic (BAME) people have a higher mortality from Covid-19 than the majority population. So far, it is unclear what the underlying reasons are. Clearly, if confirmed, these findings would be of public health relevance. Moreover, they would raise an urgent equity issue.

 Robert Aldridge and colleagues have examined the mortality of people who tested positive for Covid-19 using NHS data from hospitals in England. Based on more than 16,000 deaths they found higher numbers of deaths in BAME people and lower numbers of deaths in White British and Irish groups, when compared to the expected number based on the total population. Standardised Mortality Ratios remained significantly different after adjustment for age and geographical region.

 The statistical methodology used is transparently presented and adequate, assuming that the age distributions of the different sub-populations are broadly similar to that of the total population. Limitations of the data set are explained. The authors could have performed a sensitivity analysis with different assumptions regarding the distribution of the various ethnicities among those persons in whom this information was missing. 

 The Discussion section of the manuscript is long as compared to the Results section, mainly due to problems of interpreting the findings. I see two issues here: Firstly, “ethnicity” is socially constructed; there is no plausible biological correlate (see, for example, Saini, A. (2020). Stereotype threat. The Lancet, 395(10237), 1604-1605 ^1^). This should be explained in order to avoid speculation of underlying “genetic” causes. Second, having said that, probable underlying causes are socioeconomic disadvantages and possibly differentials in access and quality of care. The available data, however, are not really informative in this respect. In consequence, the second-but-last paragraph (starting with “We believe…”) should be shorter and less speculative.

 Minor technical issue: Some journals require reporting of confidence intervals *and* p-values (the latter are not reported in Table 1).

Is the work clearly and accurately presented and does it cite the current literature?

Yes

If applicable, is the statistical analysis and its interpretation appropriate?

Yes

Are all the source data underlying the results available to ensure full reproducibility?

Yes

Is the study design appropriate and is the work technically sound?

Yes

Are the conclusions drawn adequately supported by the results?

Partly

Are sufficient details of methods and analysis provided to allow replication by others?

Yes

Reviewer Expertise:

epidemiology; public health; health of migrants and refugees; small area differentials in health; Covid-19

We confirm that we have read this submission and believe that we have an appropriate level of expertise to confirm that it is of an acceptable scientific standard, however we have significant reservations, as outlined above.

## References

[1] : Stereotype threat. *The Lancet*.2020;395(10237) : 10.1016/S0140-6736(20)31139-9 1604-1605 10.1016/S0140-6736(20)31139-9 PMC724199132446405

# Response

**Published:** 2020-06-15

**Authors:** Robert Aldridge

**Affiliations:** UCL, UK

Thanks very much for this helpful review - we appreciate the time you have taken to carefully read and provide constructive feedback on the article. We have tried to address all the points you have raised and below we include your comments alongside in our responses ***in bold italics. ***We have numbered each of our responses in order that we can cross-reference them across all of the reviews to this article.

******

Preliminary reports indicate that Black, Asian and Minority Ethnic (BAME) people have a higher mortality from Covid-19 than the majority population. So far, it is unclear what the underlying reasons are. Clearly, if confirmed, these findings would be of public health relevance. Moreover, they would raise an urgent equity issue.

Robert Aldridge and colleagues have examined the mortality of people who tested positive for Covid-19 using NHS data from hospitals in England. Based on more than 16,000 deaths they found higher numbers of deaths in BAME people and lower numbers of deaths in White British and Irish groups, when compared to the expected number based on the total population. Standardised Mortality Ratios remained significantly different after adjustment for age and geographical region.

The statistical methodology used is transparently presented and adequate, assuming that the age distributions of the different sub-populations are broadly similar to that of the total population. Limitations of the data set are explained. The authors could have performed a sensitivity analysis with different assumptions regarding the distribution of the various ethnicities among those persons in whom this information was missing.

***Author response 6: Since the submission we have spent time exploring whether there are between-census population estimates of ethnicity that include age that would enable us to explore this issue further. Unfortunately, we have not been able to locate data that we could use for this analysis and we feel these would be required to inform a sensitivity analysis as described. We agree it would be helpful to perform this sensitivity analysis, but the current lack of data restricts our ability to do an informative sensitivity analysis and so we have not included one in this update due to the lack of available data to inform it.***

  

The Discussion section of the manuscript is long as compared to the Results section, mainly due to problems of interpreting the findings. I see two issues here: Firstly, “ethnicity” is socially constructed; there is no plausible biological correlate (see, for example, Saini, A. (2020). Stereotype threat. The Lancet, 395(10237), 1604-16051). This should be explained in order to avoid speculation of underlying “genetic” causes. Second, having said that, probable underlying causes are socioeconomic disadvantages and possibly differentials in access and quality of care. The available data, however, are not really informative in this respect. In consequence, the second-but-last paragraph (starting with “We believe…”) should be shorter and less speculative.

  

***Author response 7: We agree with the reviewers about the socially constructed nature of ethnicity and in our original submission we deliberately chose not to discuss biology or genetics because of this. In relation to this point raised by the review, we have added the following to sentence to the discussion to elaborate further in agreement with your suggestion:***

***“Ethnicity is socially constructed and correlates poorly with biology. Biological differences are therefore highly unlikely to underpin these inequalities.”***

***We believe the paragraph referred to by the authors (starting with “We believe…”) provides important and urgent public health actions that should be taken as a precautionary principle to reduce the increased mortality from COVID in BAME communities, and as such we feel it is important to restate them in this discussion. We also note the recent objections to other reports describing the inequalities in health for BAME communities that did not include recommendations such as ours on this precautionary note.***

Minor technical issue: Some journals require reporting of confidence intervals and p-values (the latter are not reported in Table 1).

***Author response 8: As suggested, we have added p-values to our updated Table 1.***

# Peer review: Reviewer response for version 1

**DOI:** 10.21956/wellcomeopenres.17464.r38664

**Published:** 2020-05-20

**Authors:** Matthew Wallace

**Affiliations:** 1 Demography Unit, Department of Sociology, Stockholm University, Stockholm, Sweden

**Summary**: This article investigates variation in the risk of death from COVID-19 across ethnic minority groups in England using *National Health Service (NHS)* data from the beginning of March to the end of April 2020. The authors produced standardized mortality ratios adjusted for age, followed by age and geographical region, to contribute new findings on the COVID-19 pandemic. The findings have public health implications and should be of interest to a wide range of audiences including the general public, other researchers, and decision makers in the United Kingdom and beyond.

**Main finding**: the authors document an elevated risk of death from COVID-19 among Black African, Black Caribbean, Pakistani, Bangladeshi and Indian ethnic minority groups. This elevated risk of death persists after adjusting for age and geographical regions.

**Comments**: the limitations of this research (which are almost exclusively data-related) are well-covered in the main text and the authors are transparent about these issues. Nevertheless, I have some suggestions and clarifications.

 First, given the number of caveats in the study, the authors should qualify their 'conclusion' part of the abstract to make clear that there are limitations in the article for those who, say, only read the abstract (of which there are more than we would like to believe).

 Second, the authors tell us that biological sex is not available but they do not go on to elaborate upon the possible consequences of its absence. Some (short) discussion around this would be welcome in light of evidence that men are more susceptible to COVID-19.

 Third, the study lacks adjustment for confounding factors such as SEP (socioeconomic position). This is simply because this information is not available with the data at hand. To offset this, the authors provide a welcome discussion of these SEP factors and how they likely vary across the different ethnic groups in England. I suspect that the sizable role that region plays in attenuating, but not eliminating, the excess risk of death in some ethnic groups is because this variable captures *some* of the excess that would otherwise be explained by these SEP factors. In any case, given the size of the some of the ethnic-specific SMRs, it is unlikely that further adjusting for SEP factors would fully equalize the substantial differences observed in the SMRs of different ethnic groups (although its presence would be welcome).

 Finally, I recommend that the assumptions made in the methods section are revisited more strongly in the discussion section. Notably, the use of denominator data that is nearly a decade old for rapidly expanding ethnic groups (is this likely to results in some over-estimation of death in the fastest-growing groups?), more explicit information on exactly how broad the age groups are (I don't think this is here, but I may have missed it), what the age range is (I don't think I see this anywhere either - is it simply all ages?), clarifying what happened to decedents with missing ethnicity (were they excluded? Combined with Other? Did you calculate the mortality of the missing group to see if the missingness was selective?), and the assumption regarding the proportion of deaths in age groups over regions (could you perhaps have used another cause of death, such as influenza, as a base instead?).

 As a final note, I wonder whether Figure 1 would be more easy to interpret if rotated (and even presented as points as opposed to bars - although this is more a personal preference). Currently, it is a little hard to read the labels and pick out specific ethnic groups.

**Final reflections:** this is an important new study (with, admittedly, several limitations) that showcases some alarming findings that demand further research.

Is the work clearly and accurately presented and does it cite the current literature?

Yes

If applicable, is the statistical analysis and its interpretation appropriate?

Yes

Are all the source data underlying the results available to ensure full reproducibility?

Yes

Is the study design appropriate and is the work technically sound?

Partly

Are the conclusions drawn adequately supported by the results?

Yes

Are sufficient details of methods and analysis provided to allow replication by others?

Yes

Reviewer Expertise:

Demography, Population Studies, Geography.

I confirm that I have read this submission and believe that I have an appropriate level of expertise to confirm that it is of an acceptable scientific standard, however I have significant reservations, as outlined above.

# Response

**Published:** 2020-06-15

**Authors:** Robert Aldridge

**Affiliations:** UCL, UK

Thanks very much for this helpful review - we appreciate the time you have taken to carefully read and provide constructive feedback on the article. We have tried to address all the points you have raised and below we include your comments alongside in our responses ***in bold italics. ***We have numbered each of our responses in order that we can cross-reference them across all of the reviews to this article. 

******

First, given the number of caveats in the study, the authors should qualify their 'conclusion' part of the abstract to make clear that there are limitations in the article for those who, say, only read the abstract (of which there are more than we would like to believe).

***Author response 1: We agree it would be useful to add a summary of the limitations to the abstract and have added the following to the conclusion:***

***“Our results were limited by the broad age groups, lack of data on deaths outside of NHS settings and denominator data on ethnicity being based on the 2011 census.”***

Second, the authors tell us that biological sex is not available but they do not go on to elaborate upon the possible consequences of its absence. Some (short) discussion around this would be welcome in light of evidence that men are more susceptible to COVID-19.

***Author response 2: To address this point we have added the following to the discussion, which also adds in comments made by the helpful review from Frances Darlington-Pollock:***

***“There is increasing evidence that men are more likely to die from COVID-19 and therefore our lack of disaggregation by sex could account for some of the remaining differences in SMRs we see between ethnic groups, particularly as those occupations found to be at higher risk include greater numbers of men working in them.”***

  

Third, the study lacks adjustment for confounding factors such as SEP (socioeconomic position). This is simply because this information is not available with the data at hand. To offset this, the authors provide a welcome discussion of these SEP factors and how they likely vary across the different ethnic groups in England. I suspect that the sizable role that region plays in attenuating, but not eliminating, the excess risk of death in some ethnic groups is because this variable captures some of the excess that would otherwise be explained by these SEP factors. In any case, given the size of the some of the ethnic-specific SMRs, it is unlikely that further adjusting for SEP factors would fully equalize the substantial differences observed in the SMRs of different ethnic groups (although its presence would be welcome).

***Author response 3: We agree with the reviewer on this point and would like to explore it further when data enabling us to do are available.***

Finally, I recommend that the assumptions made in the methods section are revisited more strongly in the discussion section. Notably, the use of denominator data that is nearly a decade old for rapidly expanding ethnic groups (is this likely to results in some over-estimation of death in the fastest-growing groups?), more explicit information on exactly how broad the age groups are (I don't think this is here, but I may have missed it), what the age range is (I don't think I see this anywhere either - is it simply all ages?), clarifying what happened to decedents with missing ethnicity (were they excluded? Combined with Other? Did you calculate the mortality of the missing group to see if the missingness was selective?), and the assumption regarding the proportion of deaths in age groups over regions (could you perhaps have used another cause of death, such as influenza, as a base instead?).

***Author response 4: We have clarified in the methods section that:***

***“All ages were used, and age ranges included were 0-19; 20-39;40-59; 60-79 and 80+.”***

***We assumed that decedents with unknown ethnicity had the same ethnicity structure as other decedents.***

***In the discussion we have added the following which we hope address other points raised:***

***“Our use of census data from 2011 is likely to result in over-estimation of mortality ratios in minority ethnic groups that have grown the fastest during this time period.”***

***Given the differing seasonal variation in influenza, and substantially different infection fatality ratios we are not sure that using this as a base instead would be helpful.***

As a final note, I wonder whether Figure 1 would be more easy to interpret if rotated (and even presented as points as opposed to bars - although this is more a personal preference). Currently, it is a little hard to read the labels and pick out specific ethnic groups.

***Author response 5: In the updated manuscript we’ve tried to increase the label size and also increase the image quality to make the image easier to read.***

Final reflections: this is an important new study (with, admittedly, several limitations) that showcases some alarming findings that demand further research.
